# Magnesium Status and Stress: The Vicious Circle Concept Revisited

**DOI:** 10.3390/nu12123672

**Published:** 2020-11-28

**Authors:** Gisèle Pickering, André Mazur, Marion Trousselard, Przemyslaw Bienkowski, Natalia Yaltsewa, Mohamed Amessou, Lionel Noah, Etienne Pouteau

**Affiliations:** 1 Plateforme d’Investigation Clinique/CIC Inserm 1405, University Hospital CHU, 63000 Clermont-Ferrand, France; gisele.pickering@uca.fr; 2 INRAE, UNH, Unité de Nutrition Humaine, Université Clermont Auvergne, 63001 Clermont-Ferrand, France; andre.mazur@inrae.fr; 3 Independent Regulatory Board for Auditors (IRBA), 91220 Bretigny-sur-Orge, France; marion.trousselard@gmail.com; 4 Department of Psychiatry, Warsaw Medical University, 02-091 Warsaw, Poland; pbienko@yahoo.com; 5 Department of General Medicine, The Yaroslavl State Medical University Institute of Postgraduate Education, 150000 Yaroslavl, Russia; yaltzewa@yandex.ru; 6 Medical Affairs Department, Consumer HealthCare, Sanofi, Gentilly, 94250 Paris, France; Mohamed.Amessou@sanofi.com (M.A.); Etienne.Pouteau@sanofi.com (E.P.)

**Keywords:** stress, magnesium, hypomagnesemia, magnesium deficiency, vicious circle, dietary intake, magnesium supplementation

## Abstract

Magnesium deficiency and stress are both common conditions among the general population, which, over time, can increase the risk of health consequences. Numerous studies, both in pre-clinical and clinical settings, have investigated the interaction of magnesium with key mediators of the physiological stress response, and demonstrated that magnesium plays an inhibitory key role in the regulation and neurotransmission of the normal stress response. Furthermore, low magnesium status has been reported in several studies assessing nutritional aspects in subjects suffering from psychological stress or associated symptoms. This overlap in the results suggests that stress could increase magnesium loss, causing a deficiency; and in turn, magnesium deficiency could enhance the body’s susceptibility to stress, resulting in a magnesium and stress vicious circle. This review revisits the magnesium and stress vicious circle concept, first introduced in the early 1990s, in light of recent available data.

## 1. Introduction

Stress, often intended as a psychological response to external stressors, has become a common issue of modern life [1]. From a neurobiology perspective, stress is an adaptive system that continuously assesses and interacts physically, physiologically, or psychosocially, with the environment. When this stress system is overloaded, negative health outcomes could result [2]. Magnesium is a fundamental nutrient, the role of which in human health is widely recognized [3]. Today, magnesium deficiency is also a common condition among the general population [4], and given its importance in the functioning of many reactions of the human body, this deficiency can increase the risk of physical and mental health illness over time. Of note, symptoms of magnesium deficiency and stress are very similar, the most common being fatigue, irritability, and mild anxiety [5,6,7]; further symptoms are shown in Table 1.

The idea of a bidirectional relationship between magnesium and stress was first introduced by Galland and Seelig, in the early 1990s [9,10] and then referred to as the vicious circle. This vicious circle implies that stress can increase magnesium loss, causing a deficiency; in turn, magnesium deficiency can enhance the body’s susceptibility to stress [10].

Taking into account the increasing prevalence of stress in modern societies [11], and its related consequences to health, this review revisits the magnesium and stress vicious circle concept, with a focus on the role of magnesium on the body’s response to stress and the pathways that regulate such a response. In particular, the scope of this article was to assess the evidence available on the need of an adequate intake of magnesium, and strengthen the hypothesis that a revision of the current recommended intake of magnesium is needed for the general population when exposed to stress, in order to reduce associated health risks.

## 2. Magnesium: Biological Role and Dietary Needs

### 2.1. Biological Role of Magnesium and Homeostasis

Magnesium is an essential mineral for humans [12]. Being the second most abundant intracellular cation [5], magnesium is involved in almost all major metabolic and biochemical processes [13]. It acts as a cofactor in hundreds of enzymatic reactions [13], its primary functions including protein and nucleic acid synthesis, regulation of metabolic pathways, neuronal transmission, neuromuscular function, and regulation of cardiac rhythm [8,14,15]. In addition, magnesium is a naturally occurring calcium channel blocker, is involved in the maintenance of electrolyte balance (e.g., regulation of sodium–potassium ATPase activity), and plays a key role in membrane excitability [5,12].

It is estimated that an adult human body contains around 21–28 g of magnesium, 50–60% of which is stored in the bones, with the remainder distributed in soft tissues such as muscles [14,16]. Magnesium is also an essential component of the extracellular fluid (ECF) and the cerebrospinal fluid (CSF) in the central nervous system [17,18]. Magnesium enters the brain through the blood–brain barrier which maintains the passage of nutrients and electrolytes for the ECF homeostasis, and is actively transported by choroidal epithelial cells into the CSF [17,18]. Although little has been revealed about the exact mechanisms of magnesium transport into the brain, it is known that magnesium concentration is higher in the CSF than in plasma [19]. Under conditions of deficiency, magnesium levels still decline in the CSF, but slower when compared to the changes observed in plasma magnesium levels [19]. Experimental studies have shown that in magnesium-deficient animals, the brain uptake of magnesium is almost doubled compared to normal-fed controls [20] and CFS magnesium concentration was readily repleted, showing that magnesium is an essential mineral for the brain homeostasis [19,20].

Only 1% of the total magnesium is extracellular and 0.3% of this circulates in serum in three different forms [21]: Free (unbound; 60%), which represents the biologically active form; albumin-bound (30%); or in a complex with other ions (10%) [13].

**Figure 1:**
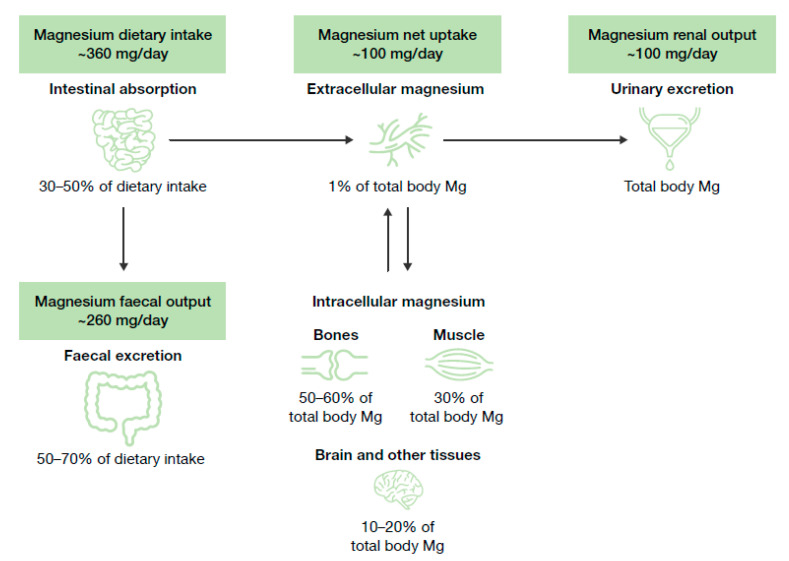
Magnesium homeostasis. Figure adapted from Jahnen–Dechent, 2012 [17]. Data from Elin, 1988 [19] and deBaaij, 2015 [8]. Mg, magnesium.

Magnesium homeostasis is tightly regulated and relies on the dynamic balance between intestinal absorption, kidney excretion, and storage in bones (Figure 1) [22]. Magnesium is mainly absorbed in the distal parts of the small intestine [22], and mostly stored in bones [22], where it serves as a reservoir to maintain the equilibrium with its extracellular concentration [22]. The kidneys play a critical part in magnesium homeostasis by eliminating its excess [22].

Many factors can affect magnesium balance: A diet high in sodium, calcium, and protein [5,23,24,25], the consumption of caffeine and alcohol [5,13,26], and the use of certain medicines such as diuretics, proton-pump, and inhibitors or antibiotics [13,26,27], which can all cause lower magnesium retention. In healthy individuals, some physiological conditions such as pregnancy [28,29], menopause [30], or ageing [31,32] are associated with changes in the need for magnesium. Pathological conditions, particularly those affecting the absorption and the elimination of nutrients (e.g., diabetes, renal function impairment, and physiological stress), may also result in significant magnesium loss or malabsorption [4,5,26,33,34]. Studies on hereditary forms of magnesium deficiency have contributed to the identification of both recessive and dominant genetic disorders directly affecting the transport of magnesium at a cellular level [35]. Although mutated transporting-proteins mainly contribute to renal wasting or intestinal malabsorption of magnesium, the mechanisms at the molecular level remain to be elucidated [8,36]. Notably, several studies showed that lower magnesium levels are involved in the course of several mental disorders, especially depression [37]. A summary of the factors affecting magnesium homeostasis is presented in Table 2.

### 2.2. Food Sources, Current Recommended Intakes and Safety

Nuts, legumes, whole cereals, and fruits have the highest magnesium content of all foods [16]. Coffee or cocoa-based products may also contain significant amounts of magnesium, while fish, meats, and milk have an intermediate amount [46,47]. Drinking water, especially harder water, can also be rich in magnesium salts [48]. The source of dietary magnesium varies widely according to gender, age, and dietary habits. For example, French adults in 2016 obtained more than 21% of their magnesium from hot beverages including coffee, 9% from bread, and 6% from vegetables [47], whereas in a sample of American adults, the main sources of magnesium were vegetables (13%), milk (7%) and meat (7%) [49]. In a sample of Polish adults, dietary magnesium requirements were mostly maintained by the consumption of cereal products (11.8–15.3%) [50], and milk or dairy products (10.9%) [51]. A study investigating the Italian diet found that cereals (27%) are the primary source of magnesium in adults [52].

Over time, public health agencies have reviewed and established recommendations for dietary intake of magnesium (and other nutrients). These include the estimated average requirement (EAR), which represents the average daily intake that satisfies the nutritional requirement of 50% of the population considered; and the recommended dietary allowance (RDA), which is the daily intake that meets the requirement of 97.5% of the same population [15,53]. Values are set on the basis of dietary balance experiments and/or results from clinical studies and meta-analyses [54,55,56,57].

Dietary balance studies performed in the 1980s in the USA concluded that the EAR for magnesium was 310–330 in men and 255–265 mg/day in women [55,56,57]. As a consequence, in 1997, the Standing Committee on the Scientific Evaluation of Dietary Reference Intakes (for the USA and Canada) set an RDA of 400–420 and 310–320 mg/day for men and women, respectively [58]. Nowadays, nutrient requirements and dietary guidelines are available in every country. For instance, in Poland, the RDA for magnesium is 400–420 for men and 310–320 mg/day for women [59], and in Russia, it is 300 mg/day for both men and women [60]. The European Food Safety Agency (EFSA) did not consider the available scientific evidence strong enough to determine RDAs, and has suggested an “adequate intake” of 350 and 300 mg/day for men and women, respectively [16]. Within the EU, national governmental bodies have set local RDAs. In 2015, the Japanese Ministry of Health, Labor and Welfare updated the dietary reference intake guidelines and set the RDA at 320–340 and 220–230 mg/day for adult men and women, respectively [61]. Recommended intakes are summarized by country in Table 3.

Studies have consistently shown that the dietary magnesium intake is often inadequate across different countries [5,64]. In 2005, King et al. reported that approximately 60% of Americans do not reach the recommended daily intake of magnesium through their diet [65]. In the USA, between 2003–2006, the average intakes of magnesium from food were 268 for men and 234 mg for women, which meant that 63% of men and 69% of women did not meet the EAR [66]. These results were confirmed and supported by the Dietary Guidelines Advisory Committee, which, in 2015, concluded that magnesium is an under-consumed nutrient for many Americans [46].

In Europe, the situation is similar. The National Diet and Nutrition Survey conducted in the UK between 2014–2016 showed that men’s mean dietary intake in magnesium was 302 and women’s was 238 mg/day [67]. In France in 2007, the mean daily dietary intake was 323 in men and 263 mg in women and more than two-thirds of the French adult population (67.4% of men and 76.7% of women, aged 18 to 54) had an inadequate magnesium intake [68]. Furthermore, in Spain, the Anthropometry, Intake and Energy Balance in Spain (ANIBES) study revealed that the mean consumption of magnesium in the population was 222 mg/day, indicating that 79% of the population had an intake below 80% of the national RDA [69]. The Mediterranean Healthy Eating, Ageing, and Lifestyle (MEAL) observational study conducted in Italy found that the dietary intake of magnesium was adequate both in men (397) and women (390 mg/day), with cereals, dairy products, and legumes being the main food sources [70]. Lastly, an analysis based on national surveys conducted across European countries showed that the mean magnesium intake among adults (18–60 years old) in Poland was 396 in men and 264 mg/day in women; [71] whereas German adults had the highest mean intake magnesium, 522 in men and 418 mg/day in women [71].

When there is a need for optimizing the magnesium status, a variety of oral supplements are available. Magnesium supplementation is considered well-tolerated, with diarrhea typically being the main manifestation of an excessive intake [72]. The upper limit for magnesium supplementation in healthy adults is 350 mg/day [73]. Normally, an increased renal filtration can reverse a wide range of serum magnesium concentration to normal levels. However, serious adverse effects have been reported for serum magnesium concentration exceeding 1.74–2.61 mmol/L. Symptoms of magnesium toxicity include hypotension, nausea, flushing of the face, retention of urine, and lethargy, and may progress to difficulty breathing, extreme hypotension, irregular heartbeat, and cardiac arrest [72].

### 2.3. Magnesium Deficiency: Causes and Health Consequences

In clinical practice, measurement of serum magnesium levels is the most common means of assessing nutrient status [13], with normal values considered to be within the 0.7ߝ1.0 mmol/L range [74,75]. Hypomagnesemia is clinically defined when serum concentrations drop below 0.7 mmol/L [8]. Severe hypomagnesemia (<0.4 mmol/L) is rare and occurs mostly in serious pathological conditions [76]. Symptoms may include neuromuscular dysfunction (muscular weakness, tremors, seizures or tetany); cardiovascular signs (electrocardiographic abnormalities and arrhythmias); and hypokalemia and hypocalcemia [13]. However, mild hypomagnesemia (0.5–0.7 mmol/L) is common and estimated to affect around 2.5ߝ15% of the population [4,26]. In the majority of cases, magnesium deficiency is not identified, as low serum levels are compensated by the release of magnesium from the bone reservoir [48]. In addition, mild deficiency can remain undetected as it often occurs with nonspecific symptoms such as irritability, nervousness, mild anxiety, muscle contractions, weakness, fatigue, and digestive troubles [26]. In addition, it has been suggested that chronic latent magnesium deficiency could start developing below 0.85 mmol/L with a potential impact on human health [12,77]. A recent study by Noah et al. found that nearly half (~44%) of the subjects screened for stress had chronic latent magnesium deficiency (defined as serum magnesium <0.85 mmol/L) [78]. Moreover, subclinical, chronic magnesium depletion may contribute to various dysfunctions and diseases and the scientific literature is rich in studies highlighting the association between low dietary magnesium intake and a higher risk of type 2 diabetes, cardiovascular diseases, osteoporosis, and metabolic syndrome [8,79,80].

Several factors contribute to magnesium deficiency (Table 2). Dietary surveys point to an inadequate magnesium intake from food. Surveys conducted in different countries have consistently showed a substantial inadequate intake of magnesium from food in the general population, particularly in young adults, those over 70 years of age [81], and in women [71]. Of note, over the past 60 years, intensive farming practices have caused a significant depletion of the mineral content of the soil [82,83,84], including a decrease in magnesium of up to 30% [85,86]. Additionally, western diets typically have a greater proportion of processed food, where several products are mostly refined, with magnesium being depleted by up to 80–90% in the process [5,8]. Factors and behaviors associated with the western lifestyle, including intense sport and physical activity [38], poor sleep quality and quantity [41], and psychological stress [43,44], can also induce magnesium loss. Magnesium deficiency is linked to many health conditions, from those affecting its metabolism, such as gastrointestinal diseases, type 2 diabetes, alcohol dependence, or kidney failure [5,26], to genetic disorders [35]. A growing body of evidence also suggests that chronic stress may cause magnesium loss/deficiency [43]. Numerous studies have shown lower magnesium levels associated with different neurological and psychiatric disorders, particularly depression and post-traumatic stress disorder [87,88] but also anxiety disorders, attention deficit hyperactivity disorder, and bipolar disorder [37,89]. Although evidence on a causal factor between mental disorders and magnesium deficiency has yet to be confirmed, stress appears as a key component in the relationship between mental health illness and magnesium deficiency.

## 3. Stress

Stress is commonly described as a trigger that evokes a physiological and psychological response of the body [90]. Over the past decades, the understanding of stress biology has largely evolved. Stress is no longer considered as a temporary response to occasional threats, but rather an ongoing and adaptive system that enables an individual to assess, cope, and predict constantly changing conditions. However, the capacity of this stress system is limited and can be overloaded, resulting in poor health outcomes, particularly those related to mental illness like depression or cognitive deficits [2].

Stress not only affects the mental health status of an individual, but it is also characterized by a physical response of the body that, depending on the type and length of exposure, may lead to short-term effects (e.g., increased blood pressure, increased heart and respiration rates, increased alertness) [91], or long-term effects (e.g., impaired hippocampal neurogenesis, cognitive and memory disorders) [92].

The following sections summarize widely recognized theoretical models of stress and describe possible physiological roles of magnesium in the stress response. Here, the term “stress model” refers to a theoretical framework used to predict outcomes and to explain specific processes.

### 3.1. Neurobiological Stress and Allostatic Load Model

In the 1950s, Selye proposed the general adaptation syndrome model to describe stress as the reaction of the body to emergency situations [93]. This theory divides the response to stressful stimuli into three phases: (1) Alarm: Upon perceiving a stressor, the body reacts with a “fight-or-flight” response, the sympathetic nervous system (SNS) is stimulated, and the body’s resources are mobilized to meet the threat. (2) Resistance: The body resists and compensates as the parasympathetic nervous system (PNS) attempts to return many physiological functions to normal levels, while the body remains on alert and focuses resources against the stressor. (3) Exhaustion: If the stressor continues beyond the body’s capacity, the resources are depleted and the body becomes susceptible to disease (distress) [91,93].

A more contemporary concept, which better describes the cumulative impact of stressor exposure on health outcomes, is that of allostasis. Allostasis is the process by which constant changes allow an organism to achieve and maintain normal functions, thus reflecting the ability of the body to adapt to daily situations like exercising or hunger, effectively [94]. However, this continual maintenance costs the body energy and resources, and over time, may lead to symptoms of allostatic load—the functional and structural damage caused by “the wear and tear” of the body’s resources in response to stress [95]. Therefore, the response to a new stressor depends on the body’s resources available following the previous stress response [96]. The allostatic load is characterized by a cumulative effect, which becomes greatest when stress is chronic or intense [96].

The hypothalamic–pituitary–adrenal (HPA) axis and the autonomic nervous system (comprising SNS and PNS) have been identified as the mediators of this neurobiological stress model [90,91,95]. First, corticotrophin-releasing factor (CRF) is secreted from the paraventricular nucleus in the hypothalamus; the subsequent secretion of adrenocorticotropic hormone (ACTH) from the anterior pituitary stimulates the release of glucocorticoids (mainly cortisol) from the adrenal cortex [97]. Noradrenaline (NA) and adrenaline are also released from the sympathetic nerves and the adrenal medulla, and together with the glucocorticoids regulate the stress response [90,91]. Cortisol also interacts with the serotonergic pathway, adjusting the release of serotonin (5-hydroxytryptamine or 5-HT) neurotransmitter in response to acute or chronic stressors [98]. Serotoninergic neurons modulate the stress response either via direct neurotransmission to the hypothalamus, or by stimulation of noradrenergic neurons [97]. In addition to the regulation through feedback mechanisms, the HPA axis is also modulated by other central systems, particularly by the inhibitory action of the γ-aminobutyric acid (GABA), and the excitatory effect of glutamate [99].

In this neurobiological model, cortisol is a well-known mediator of the stress response. The nocturnal cortisol urinary excretion in apparently healthy subjects reflects the basal tone of the HPA axis [100]; conversely, the blood cortisol concentration measured in a challenging environment is a sign of stress activity [101]. It has been shown that cortisol coordinates the central response to stress at several levels [102], and indirectly influences mechanisms of neuroprotection [103]. Neurotrophic factor production, represented by the brain-derived neurotrophic factor (BNDF), intervenes in allostasis through protecting neurons [104]. Normally, BNDF promotes neuronal survival and plasticity [104]; however, changes in BNDF expression have been reported following exposure to stressful stimuli. An increase of BNDF has been observed in response to moderate stress [105], whereas a decrease has been associated with high levels of stress [106]. Furthermore, increasing evidence shows a link between cortisol responses and oxidant elevation [107]. The accumulation of free radicals and other reactive oxygen species is also a sign of allostatic load, resulting from the imbalance between cellular metabolic activities and antioxidant defense mechanisms [108,109].

Noteworthy, magnesium interacts with all these stress mediators [17,110,111,112], overall serving an inhibitory function in the regulation and central neurotransmission of the stress response (details of these interactions are summarized in chapter 6).

### 3.2. Generalized Unsafety Theory of Stress (GUTS) Model

Conventional theories of stress have historically focused on the assumption that stress is a response to an actual environmental threat (either internal or external to the body), making it difficult to explain the relation between stress and disease. In contrast, GUTS is a new psychological and cognitive theoretical model proposed by Brosschot in 2016 [113] that revises and expands the stress theory by focusing on safety instead of threat, and by including risk factors that have hitherto not been attributed to stress [113]. Based on neurobiological and evolutionary evidence, GUTS hypothesizes that stressors are not necessary for a chronic stress response to occur but the perception of an unsafe state is enough. In GUTS, PNS is the key system controlling the stress response (particularly the vagus nerve and the prefrontal cortex activity) [113]. Of note, preclinical data suggest that magnesium may be important for the functionality of these central systems. An excess of magnesium or magnesium deficiency have been shown to modulate the autonomic nervous system, but further research is still needed [114,115,116].

The GUTS model suggests that the default stress response can be chronically activated in various situations, three of which are particularly susceptible to health risks. (1) Reduced body capacity: In compromised physical conditions, e.g., obesity or aging, the brain perceives the body as inadequate to be able to “fight-or-flight”, and therefore maintains a state of general alarm, or unsafety [117]. (2) Compromised social network: Being part of a group is a fundamental aspect of survival for social animals and humans, and isolation is one of the main conditions in which safety is lacking [117]. Interestingly, there is evidence that patients suffering from metabolic syndrome [118] or congestive heart failure [5] (both conditions of reduced body capacity as described in GUTS) exhibit lower serum magnesium concentration. (3) Perceived aversive environment: In cases of specific stressors (e.g., work stressors), a neutral daily environment (e.g., an office working environment) can be perceived as unsafe [117]. The GUTS model suggests that repetitive negative thinking may result in the impairment of key systems controlling the stress response [119]; however, the relationship between general unsafety and magnesium status is to be elucidated yet.

## 4. Evidence of the Impact of Stress on Magnesium Homeostasis

Initially, the shift from intracellular to extracellular magnesium following a stressor exposure plays a protective and regulatory role [90]. Normally, magnesium inhibits the glutamatergic transmission while promoting GABA activity, resulting in a mostly inhibitory effect at the central level [42]. Magnesium also tends to diminish the stress response mediated by catecholamines and glucocorticoids. However, a chronic stressor exposure may result in a depletion of various resources as described by Selye, including magnesium [42,93]. The progressive loss of magnesium from the reservoir in bone can eventually compromise its physiological inhibitory action and lead to an over-activation of the HPA axis and neuronal hyperactivity [120]. The impact of stress on magnesium status has been extensively investigated in both animal and human studies [42,121].

***Pre-clinical evidence.*** Animal studies have shown a transient hypermagnesemia in the short-term period after the exposure to acute stress stimuli [122,123,124,125]. A series of experiments conducted on cats by Classen et al. showed that stimuli such as withdrawal of blood, infusion of catecholamines, or potassium poisoning all caused an increase of blood magnesium concentration [122]. This increase was not influenced by pre-treatment with an adrenergic blocking agent (e.g., reserpine), suggesting that other mechanisms rather than catecholamines are responsible for the change in magnesium levels [122]. Similarly, a shift of magnesium from erythrocytes to serum was reported in different studies investigating the effect of acute noise on magnesium-deficient guinea pigs [123] and rats. As a consequence, a net renal excretion of magnesium occurs, leaving the animal magnesium deficient.

Mild hypomagnesemia can be observed in response to mid- or long-term exposure to stress. A study conducted on guide dog candidates at different levels of a training program (elementary, intermediate, and advanced) showed the effects of temperature and physical stress on serum magnesium levels. First, it was demonstrated that serum magnesium levels were significantly lower in winter than in summer (average temperature was 6 and 29 °C, respectively), suggesting an impact of seasonality on magnesium homeostasis. Thereafter, it was noticed that physical exercise had a greater impact on serum magnesium levels of dog candidates in the elementary class compared to more trained ones. These results were lastly confirmed by a third experiment, assessing both the impact of physical stress and temperature on serum magnesium levels and finding that serum magnesium levels after exercise were significantly lower in winter than in summer. [126]. The impact of physical stressors was assessed also in another study conducted on rats. A greater serum magnesium reduction was observed in those administrated with ethanol and then exposed to restraint stress, compared to control rats facing the same restraint test but receiving water [127]. An additional study conducted by Heroux et al. on rats fed with a magnesium-deficient diet and kept at low temperature (6 °C) for about 17 months found that the studied animals were capable of adapting to cold stress despite suboptimal magnesium intake; initial signs of magnesium deficiency (including skin sores, reduced growth rate, lower levels of magnesium in most organs) gradually disappeared after two months. However, regardless this adaptation, the long-term stress resistance (measured as cold resistance at −20 °C) of magnesium-deficient rats was reported to decline over time when compared to controls [128]. Lastly, exposure to cold (2–5 °C) and a deficient dietary intake of magnesium significantly reduced plasma magnesium in sheep, whereas no effect was observed in normally fed sheep [129].

***Clinical evidence.*** To help elucidate the stress hormone-induced magnesium deficiency and its clinical relevance, Whyte et al. investigated the effect produced by the infusion of adrenaline on plasma magnesium concentrations [130]. They found that magnesium levels were significantly reduced not only during the infusion time but also an hour after test cessation, without any sign of recovery [130]. A variety of tests have demonstrated that magnesium levels, both in serum and urine, are affected by the exposure to stress stimuli. Significant reductions in plasma and total magnesium concentrations were reported in a 3-month analysis on young adults exposed to either chronic or sub-chronic stressful conditions (e.g., acts of intolerance or fear of military actions) [43]. A similar effect was also seen in a study investigating the effect of temporary (one day) and chronic (one month) sleep deprivation on magnesium levels; in a group of otherwise healthy men, chronic sleep restriction was associated with greater reductions in erythrocyte magnesium concentrations [131]. University students during an exam period reported an increase in anxiety that was also associated with an increased urinary excretion of magnesium [44]. In a similar study conducted on college students during the 4 weeks following an examination period, erythrocyte magnesium content was found to be significantly depleted [132]. Interestingly, the variations in blood and urine magnesium levels were confirmed by Mocci et al. who studied the effect of noise on catecholamines and magnesium serum and urinary excretions on healthy men [133]. Mocci and his study group also noted how the timing for the change to occur was very different between the two variables, with serum magnesium increasing a few hours after the exposure to the noise (probably reflecting extracellular flux immediately after the stress), and urine excretion reaching a peak in a few hours but lasting up two days [133]. A similar result was reported by Ising et al. on a study investigating the effect of traffic noise on workers’ performance. Under noise stress (7 h), a decrease in erythrocyte magnesium levels was observed, followed by an increase of serum levels and urine excretion of magnesium [134]. The impact of acute stress on transient hypermagnesemia was noted also under physical stress. Short- and long-term exercise (20 min versus 1 h, respectively) had a different influence on the plasma magnesium levels: an increase of plasma magnesium was reported after short-term exercise but not after long-term exercise. However, after both physical tests, magnesium levels dropped below the pre-exercise values [135]. A summary of the pre-clinical and clinical evidence is shown in Table 4.

## 5. Evidence of the Impact of Magnesium Status on Stress Susceptibility

***Pre-clinical evidence.*** The relationship between magnesium deficiency and stress-related behavior is well documented. In 1986, Caddell et al. reported an increase of circulating catecholamines following the exposure of magnesium-deficient rats to a noise stress test [136]. The relationship between low serum magnesium concentrations and the increased release of catecholamines in the central nervous system was then confirmed in studies conducted on mice selected for low (MGL) and high (MGH) blood magnesium. The simple selection of genetic traits inducing low blood magnesium was found to significantly affect the metabolism of NA but not that of other neurotransmitters [137]. MGL mice not only showed higher NA levels (17% in the brain; 200% in urine) but also a more restless behavior and higher rectal temperature, all signs of an exaggerated stress response [138]. In a different study also conducted on MGL and MGH mice, both fed with the same magnesium-rich diet, the number of stress-induced gastric ulcers through the immobilization test was higher in the magnesium-deficient mice [139]. Besides the noradrenergic hyperactivity in basal magnesium deficiency conditions, dietary magnesium restrictions have also been associated with an upregulation of the stress system via increases in CRH and ACTH levels [140]. Experimental data indicate that magnesium-deficient rats exhibit more anxiety- and depression-like behavior compared with controls [42,138]. For example, in the light–dark test (often used to screen for anxiolytic and antidepressant drugs) [141], mice with magnesium deficiency showed a net preference for the darker compartment [42,140,141]. Similarly, in the forced swimming test, magnesium-deficient rats spent more time immobile compared with their controls [142,143]. Dietary magnesium deficiency in laboratory animals was also associated with stress-like behavior in the open field test. Rats with a reduced dietary magnesium content tended to visit the bright and central area less frequently [142,143], even when motivated by the presence of food, showing a psychological stress caused by the open space [140].

***Clinical evidence.*** Results in human studies are consistent with animal findings and show low magnesium status in stressed/depressed populations. In a study investigating the potential benefit of magnesium supplementation in Russian women who suffered from chronic emotional stress, Akarachkova et al. found that at baseline the majority of women were suffering from symptoms like irritability, fatigue, and sleep disorders, and 60% presented magnesium deficiency [144]. In other studies, subclinical chronic magnesium deficiency was found in up to 45% of the stressed subjects enrolled [78,145,146]. Nielsen et al. found that out of 96 American adults complaining of sleep disorders, a potential source of stress, 58% were consuming less than the EAR for magnesium and had higher levels of C-reactive protein (CRP), an indicator of inflammatory stress [145]. Lastly, it is generally recognized that chronic stress and magnesium deficiency may influence an individual’s susceptibility to depressive disorders [147]. In a group of Australian patients experiencing depression and/or anxiety, the analysis of their nutrition status showed that 22% of participants did not meet magnesium EAR. Furthermore, magnesium intake (expressed as % EAR) was negatively correlated with stress, depression, and total Depression Anxiety Stress Scale (DASS) scores [148].

A summary of the pre-clinical and clinical evidence supporting a relation between magnesium status and stress is shown in Table 5.

## 6. Proposed Model for the Vicious Circle of Stress and Magnesium Deficiency

Over the years, a growing body of evidence has consistently shown that magnesium acts on several key physiological steps involved in the response to stressful stimuli.

**Figure 2:**
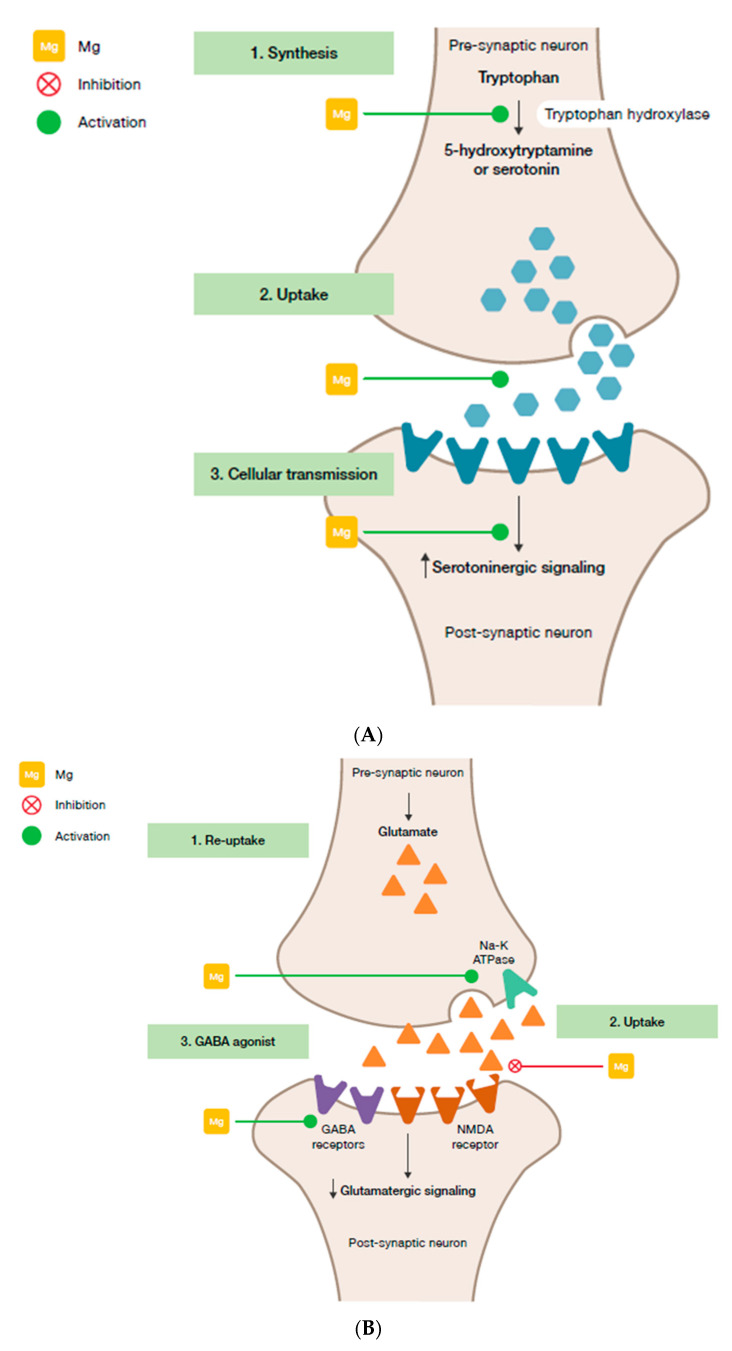
Interaction of magnesium and neurological stress mediators. (**A**) Serotoninergic transmission. (**B**) Glutamatergic and GABAergic transmission. GABA, γ-aminobutyric acid; Mg, magnesium; NMDA, N-methyl-D-aspartate.

- Magnesium and HPA. *5-HT transmission:* Magnesium directly enhances the interaction between 5-HT and its membrane receptor, and it promotes the cellular transmission of the serotoninergic signal (Figure 2A) [90]. Additionally, magnesium is a cofactor of tryptophan hydroxylase, the enzyme involved in 5-HT synthesis [90]. Glutamatergic transmission: Magnesium inhibits the glutamate directly and indirectly by blocking the glutamate N-methyl-D-aspartate (NMDA) receptor and by enhancing its reuptake in the synaptic vesicles through stimulation of the sodium–potassium ATPase, respectively (Figure 2B) [42]. GABA transmission: A GABA-agonistic activity of magnesium has been observed, although the mechanism has not yet been elucidated, (Figure 2B) [42]. *Cortisol:* Magnesium indirectly reduces the release of ACTH by modulating the neurotransmission pathways, and therefore decreases cortisol levels in the body [42];
- Magnesium and neuroprotection. Studies on the antidepressant effects of magnesium have shown the positive impact of this mineral on the expression of BNDF in the brain [149,150];
- Magnesium and oxidative stress. Magnesium may be involved in suppressing the production of free radicals in various tissues including the brain [17], and several laboratory studies have shown that magnesium-deficient animals are more at risk of oxidative stress [112,151].

**Figure 3:**
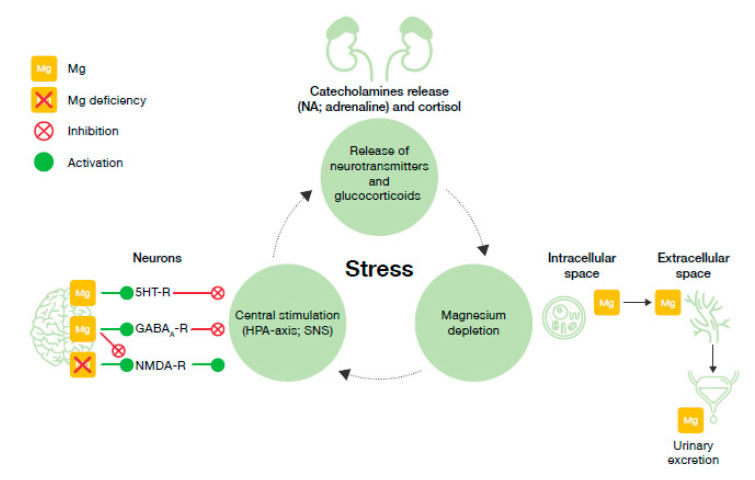
The vicious circle of stress and magnesium. GABAA-R, γ-aminobutyric acid-A receptor; Mg, magnesium; NMDA-R, N-methyl-D-aspartate receptor; NA, noradrenaline; SNS, sympathetic nervous system, 5HT-R, 5-hydroxytryptamine receptor.

In response to a stressful stimulus, stress hormones are released, causing an increase of magnesium extracellular levels [90]. As a consequence, higher magnesium concentrations are excreted through the kidneys [133]. When the stressor persists over time, this mechanism may contribute to magnesium cation depletion and deficiency [42,130], and trigger the stress and magnesium vicious circle as illustrated in Figure 3.

Comprehensively, both pre-clinical and clinical studies’ results point to the bi-directional relationship between magnesium levels and stress: Magnesium deficiency can induce symptoms and increase susceptibility to stress, and acute and chronic stress can precipitate magnesium deficiency [10,104,106].

## 7. Magnesium Supplementation

Magnesium supplementation has proven benefits for the treatment of symptoms of psychological daily stress (fatigue, irritability, sleep) [144]. It has been shown that subjects with mental and physical stress can benefit from a daily intake of magnesium. Male students experiencing common stress factors such as sleep deprivation, malnutrition, and a lack of physical activity, and receiving magnesium 250 mg/day for four weeks not only presented an increase in erythrocyte magnesium content but also a reduction of serum cortisol [152]. Magnesium supplementation of 400 mg/day was associated with a clear improvement of the heart rate variability, measured as an indicator of the parasympathetic and vagal systems’ response to stress, in subjects who were asked to complete moderate muscle endurance training once weekly [153]. The daily supplementation with 300 mg (combined or not with vitamin B6, 30 mg) provided positive results on stress relief [154], particularly on subjects who reported severe stress levels at baseline, with a reduction in Depression Anxiety Stress Scale scores of up to 45% from baseline [154]. It is interesting to note that several studies investigating the potential benefit of magnesium supplementation in populations with symptoms of stress reported a subclinical chronic magnesium deficiency or a low magnesium status at baseline in the majority of the subjects enrolled [37,78,144,145,146]. Nevertheless, despite several studies reporting an association between magnesium deficiency and stress, the effect of magnesium supplementation on stress has been less documented than its effects on depression [37,155] and anxiety disorders [156]; therefore, further investigation is still needed on stress symptoms. A possible limiting factor to the performance of such studies could be the difficulty of setting up optimal experimental conditions for studying the effect of stress; however, this challenge may be overcome in future analyses by focusing on well-defined conditions (e.g., psychological stress), and by using robust and validated tools to assess stress (such as DASS scores).

## 8. Conclusions: Implications in Terms of Dietary Magnesium Needs

Over the past decades, increasing evidence, as shown in the present narrative review, has investigated and supported the link between magnesium deficiency and increased susceptibility to stress disorders, and further suggested that stress itself can lead to magnesium depletion. Magnesium is an essential element involved in reactions regulating the body’s stress response at several levels. Severe magnesium deficiency is rare, but chronic latent deficiency appears to be common among the general population and even more among those suffering from a number of chronic diseases or stress [5]. Although the current intake of magnesium through our diet seems sufficient to avoid overt signs of magnesium deficiency in the majority of the population, it might not be adequate to provide optimal health and risk reduction of chronic diseases [5]. Stress is also an increasing condition worldwide and its effects can negatively impact health outcomes. Noteworthy, magnesium intake has been found negatively correlated with subjective stress in some populations [148], and magnesium supplementation has shown benefits in stressed but otherwise healthy subjects [153,154]. Additionally, magnesium intake is safe with limited side-effects in cases of chronic overconsumption [72].

To conclude, while there is good evidence from animal and human studies of the bi-directional link between magnesium and stress, further research is needed to better understand the impact of this correlation and the benefit of magnesium supplementation on general health. Additional studies should apply standard methodologies (e.g., magnesium load test) to evaluate the magnesium status in well-characterized stressed population. These studies would help to demonstrate the increased need of magnesium supplementation during stress periods, and further strengthen our initial hypothesis. Further, in line with the GUTS model, repetitive negative thinking could be considered as a cognitive indicator of stress and evaluated in relation to blood magnesium levels in a cohort of subjects exposed to chronic stress. Given the strong association of stress with mental and physical diseases, these studies are fundamental to further support adequate magnesium dietary needs.

## Figures and Tables

**Table 1:** Symptoms of magnesium deficiency and symptoms of stress.

Most Frequently Reported Symptoms of Stress [6,7]	Symptoms of Magnesium Deficiency [5,8]
**Fatigue**	**Tiredness**
**Irritability or anger**	**Irritability**
**Feeling nervous**	**Mild anxiety/nervousness**
**Lack of energy**	**Muscle weakness**
**Upset stomach**	**Gastrointestinal spasms**
**Muscle tension**	**Muscle cramps**
**Headache**	**Headache**
Sadness/depression	Mild sleep disorders
Chest pain/hyperventilation	Nausea/vomiting

Note: Similar symptoms are highlighted in bold.

**Table 2:** Factors contributing to magnesium deficiency.

**Diet related**
Inadequate magnesium intake [5,13,26]
High protein diet [5,25]
High sodium diet [5,25]
High calcium diet [5,23,24,25]
High caffeine intake [5]
Alcohol dependence [5,13,26]
**Lifestyle**
Sports [25,38,39,40]
Sleep quality and quantity [41,42]
Chronic stress [43,44]
**Pharmacological related**
Diuretics, e.g., furosemide [13,26,27]
Proton-pump inhibitors, e.g., omeprazole [13,27]
Cisplatin [13,26,27]
Antibiotics, e.g., gentamicin [13,27]
**Physiological conditions**
Pregnancy [28,29]
Ageing [31,32]
Menopause [30]
**Pathological conditions**
Genetic disorders [8,35,36]
Type 2 diabetes mellitus [4,5]
Gastrointestinal disorders [5,26]
Kidney failure [5,33]
Cardiovascular diseases [5,45]
Metabolic syndrome [5,34]
Osteoporosis [15,25,33]

**Table 3:** Current magnesium recommended dietary allowances (RDAs) across countries.

Country	Magnesium, mg/day	
	Men	Women
Italy [62]	240	240
Russia [60]	300	300
Japan [61]	320–340	220–230
Poland [59]	400–420	310–320
USA and Canada [58]	400–420	310–320
France [63]	420	360

Values shown refer to adult population only (≥19 years).

**Table 4:** Summary of the pre-clinical and clinical evidence supporting the impact of stress on magnesium homeostasis.

Evidence of the Impact of Stress on Magnesium Homeostasis			
	Population Tested	Stress Stimulus	Impact on Magnesium
Pre-clinical	Cats (*N* = 30)	Withdrawal of blood; infusion of catecholamines; potassium poisoning	↑Blood Mg [122]
	Guinea pigs (41)	Noise	↑Serum Mg, ↓Erythrocytes Mg [123]
	Rats (88)	Noise	↑Serum Mg, ↓Erythrocytes Mg [124]
	Rats	Noise	↓Serum Mg, ↓Erythrocytes Mg
	Dogs	Physical exercise, temperature	↓Serum Mg [126]
	Rats	Ethanol/Restraint stress	↓Serum Mg [127]
	Rats	Cold	↓Tissue content of Mg [129]
	Sheep	Dietary Mg restriction, cold	↓Plasma Mg [129]
Clinical	Adults (*N* = 8)	Adrenaline infusion	↓Plasma Mg [130]
	Young adults (*N* = 35)	Chronic or sub-chronic psychological stress	↓Plasma Mg [43]
	Healthy men (*N* = 16)	Chronic sleep deprivation	↓Erythrocyte Mg [131]
	Young adults (*N* = 35)	University exams	↑Urinary Mg [44]
	Young adults (*N* = 30)	University exams	↓Erythrocyte Mg [132]
	Young adults (*N* = 25)	Noise	↑Urinary Mg ↑Serum Mg [133]
	Healthy men (56)	Noise	↑Serum Mg, ↓Erythrocytes Mg; ↑Urinary Mg [134]
	Healthy men	Short- and long-term physical exercise	↑Plasma Mg [135]

Mg, magnesium; ↑, increase; ↓, decrease.

**Table 5:** Summary of the pre-clinical and clinical evidence supporting magnesium status on stress susceptibility. ^a^ Only symptoms shown in ≥70% of women at baseline are reported.

Evidence of the Impact of Magnesium Status on Stress Susceptibility				
	Population Tested	Mg Status	Stress Stimulus	Impact on Stress Mediator/Stress
Pre-clinical	Rats (*N* = 84)	Mg-deficient	Noise	↑Catecholamines (NA, adrenaline, dopamine) [136]
	Mice (*N* = 120)	Mg-deficient	Genetic selection	↑NA [137]
	Mice (*N* = 80)	Mg-deficient	Genetic selection; forced swimming test; four-plate test	↑NA [138]
	Mice (*N* = 100)	Mg-deficient	Genetic selection; immobilization test	↑Gastric ulcers [139]
	Mice (*N* = 20/test)	Dietary Mg restriction	Hyperthermia; open field test; light/dark test; hyponeophagia test	↑CRH; ↑ACHT [140]
	Mice	Mg-deficient	Light/dark test	Depression-like behavior [42,140]
	Rats	Dietary Mg restriction	Forced swimming test	Depression-like behavior [142,143]
	Rats	Dietary Mg restriction	Open field test	Stress/anxiety [142,143]
Clinical	Women (*N* = 100)	Mg-deficient	-	Chronic emotional stress; irritability; fatigue; sleep disturbance; headache ^a^ [144]
	Adults (*N* = 264)	Mg-deficient	-	Severe stress [78,145,146]
	Adults (*N* = 100)	Mg-deficient	Poor sleep quality	↑CRP [145]
	Adults (*N* = 109)	Mg-deficient	-	Depression/anxiety [148]

ACHTH, adrenocorticotropic hormone; CRH, corticotrophin-releasing hormone; CRP, C-reactive protein; Mg, magnesium; NA, noradrenaline; ↑, increase.
